# TRANSLATION, CULTURAL ADAPTATION, AND EVIDENCE OF INSTRUMENT VALIDITY FOR A MORPHOLOGICAL EXAMINATION PERFORMED IN CHILDREN WITH AUTISM SPECTRUM DISORDER

**DOI:** 10.1590/1984-0462/2020/38/2018318

**Published:** 2020-01-13

**Authors:** Thais Arbocese Zanolla, Eduardo Perrone, Rodrigo Ambrosio Fock, Daniela Bordini, Helena Paula Brentani, Ana Beatriz Alvarez Perez, Decio Brunoni

**Affiliations:** a Universidade Federal de São Paulo, São Paulo, SP, Brazil.; b Universidade de São Paulo, SP, Brazil; c Universidade Presbiteriana Mackenzie, SP, Brazil

**Keywords:** Autism spectrum disorder, Congenital abnormalities, Body dysmorphic disorders, Physical examination, Transtorno do espectro autista, Anormalidades congênitas, Transtornos dismórficos corporais, Exame físico

## Abstract

**Objective:**

For every 100 random children diagnosed with autism, at least 20 have morphological abnormalities, often associated with syndromes. Brazil does not have a standardized and validated instrument for morphological physical examination. This study aimed to translate into Brazilian Portuguese and culturally adapt the clinical signs described in the Autism Dysmorphology Measure, as well as validate the instrument in a sample of children with autism.

**Methods:**

The original instrument was translated, culturally adapted, and published in full, following traditional procedures for translation, back-translation, and terminology adaptation according to the *Nomina Anatomica*. The sample included 62 children from a published multicenter study, with intelligence quotient between 50–69, of both genders, with chronological age between 3–6 years. Two clinical geneticists performed the morphological physical examination, which consisted of investigating 82 characteristics assessing 12 body areas. We used Cohen’s Kappa coefficient to evaluate the agreement between the two observers.

**Results:**

The final version of the instrument – translated into Brazilian Portuguese and culturally adapted – showed high agreement between the two observers.

**Conclusions:**

The translated instrument meets all international criteria, and minor anomalies and their clinical descriptions were standardized and are recognizable for physicians not specialized in genetics.

## RESUMO

**Objetivo:**

Entre 100 crianças, não selecionadas, com diagnóstico de autismo, pelo menos 20 apresentam anomalias morfológicas, quase sempre associadas a síndromes. Não há no Brasil instrumento de exame físico morfológico padronizado e validado. O objetivo foi traduzir para o português do Brasil e adaptar culturalmente os sinais clínicos descritos no Autism Dysmorphology Measure, assim como procurar evidências de validade quando aplicado a uma amostra de crianças com autismo.

**Métodos:**

Foram feitas a tradução e a adaptação cultural do instrumento original, publicado na íntegra. Foram adotados os procedimentos tradicionais de tradução, retrotradução e adaptação da terminologia segundo a *Nomina Anatomica*. Foram incluídas na amostra 62 crianças com quociente de inteligência entre 50 e 69, de ambos os sexos, com idade cronológica entre três e seis anos, provenientes de estudo multicêntrico com os procedimentos metodológicos já publicados. O exame físico morfológico foi realizado por dois médicos geneticistas e consistiu na pesquisa de 82 características que avaliam 12 áreas corporais. Para avaliar a concordância entre os dois observadores foi utilizado o coeficiente *Kappa* de Cohen.

**Resultados:**

A versão final do instrumento traduzido e adaptado culturalmente ao português do Brasil mostrou alta concordância entre os dois observadores.

**Conclusões:**

O instrumento traduzido preenche todos os critérios propostos internacionalmente e o reconhecimento das anomalias menores e sua descrição clínica estão padronizados e são de fácil reconhecimento aos médicos não especialistas em genética.

## INTRODUCTION

Autism Spectrum Disorder (ASD) is characterized by persistent deficits in communication and social interaction, as well as restricted and repetitive patterns of behavior, interests, or activities. The symptoms must be present early in the developmental period.^1^ Approximately one in every 58 children are diagnosed with ASD.^2^ Understanding the genetics of autism has not been an easy task, given the clinical and etiological heterogeneity of the disorder.

Clinical morphology allows the identification of individuals whose structural development was interrupted during early embryogenesis.^3^ The role of clinical morphology in defining the hundreds of syndromes that cause intellectual disabilities also suggests that detailing the morphological examination could be useful in autism.^4,5^ In fact, the phenotypic variability present in a significant percentage of individuals with autism could be used to detect patients with etiologically distinct ASD. Some studies have proposed that part of the children with autism could be identified by physical characteristics depicting abnormal processes that occurred during embryogenesis, dividing them into two subgroups: essential autism and complex autism.^4,5^

The essential autism subgroup has a higher recurrence rate among siblings (4 versus 0%), a larger number of relatives with autism (20 versus 9%), and a greater man/woman ratio (6.5:1 versus 3.2:1). The etiological model that explains essential autism is multifactorial or of complex inheritance. This model presupposes genetic, epigenetic, and environmental causes.^6^ The complex autism subgroup presents individuals with evidence of abnormalities that occurred at the onset of morphogenesis, expressed by significant anomalies (dysmorphisms) or microcephaly.^5^ It comprises 20% of the population studied and individuals classified in this group showed worse outcomes compared to the essential autism subgroup: lower intelligence quotients (IQs), seizures, and abnormalities in the electroencephalography (46 versus 30%) and brain magnetic resonance imaging (28 versus 13%).^5^ In this group, as a rule, patients have a syndromic condition, with the identification of causal factors directly responsible for phenotypic, genetic, or environmental aspects.^5^

Miles et al.^7^ found and validated morphological signs to be used in the classification of subgroups of individuals with autism by physicians who are not specifically trained in morphological evaluation. In the present study, the morphological physical examination is based on the Autism Dysmorphology Measure (ADM), and the definition of dysmorphic signs follows the London Dysmorphology Database (LDDB).^8^ ADM is semi-structured, easy to perform, since the physical examination does not require removing the child’s clothes, and enables the professional not specialized in genetics to identify distinct ASD subgroups phenotypically and classify them as dysmorphic or non-dysmorphic. This definition will guide the etiological research in the dysmorphic group. Higher morbidity is also expected among these children.

This study aimed to translate into Brazilian Portuguese and culturally adapt the clinical signs described in the ADM, as well as validate the instrument in a sample of individuals with autism.

## METHOD

The original instrument is published and not copyrighted.^7^ The translation and cultural adaptation followed the traditional procedures adopted for health instruments: translation, evaluation of the initial translation (back translation), and review by the expert committee.^9^

Three clinical geneticists, experts in the evaluation of morphological phenotype (expert committee), translated the instrument into Portuguese. An anatomist adjusted the document according to the *Nomina Anatomica*.^10^ A dental surgeon was consulted to clarify doubts regarding the placement, shape, and number of primary and permanent oral elements. A Brazilian clinical geneticist, also expert in dysmorphology, working in a general hospital in the United States, fluent in English, and with no knowledge of the original instrument translated the committee version into English (back translation). The expert committee held several face-to-face meetings, producing a preliminary version. The assessment of reference equivalence consisted of comparing the back-translated version with the original. An external evaluator, whose native language is Portuguese and who is fluent in English, assessed the literal correspondence between the terms of the original and back-translated versions, classifying them into similar, close, or different. The cultural adaptation of the content was evaluated by general equivalence and involved a committee with 6 candidates who took a test with 40 multiple choice questions based on pictures of 40 randomly selected morphological signs out of 82. After the test, the expert committee assessed the questions with low agreement and reviewed the terminologies that were closer to the description of dysmorphic signs in the Brazilian cultural environment. Two clinical geneticists, experts in evaluation of morphological phenotype, who did not belong to the expert committee, independently administered this version of the instrument to a sample of 62 children with ASD. After concluding these steps, the final version of the morphological physical examination for patients with ASD in Brazilian Portuguese was ready.

The sample for validation included 62 children diagnosed with ASD, of both genders, with chronological age between 3–6 years and 11 months. This (convenience) sample belongs to a multicenter study carried out in three clinics specialized in ASD in the city of São Paulo, Brazil: Universidade Federal de São Paulo (UNIFESP, Clinic of Social Cognition – Autism Spectrum Disorder Marcos Mercadante – TEAMM), Universidade de São Paulo (Autism Spectrum Disorder Program – PROTEA), and Universidade Presbiteriana Mackenzie (TEA-MACK Clinic), during the months of February and November 2014. Its main inclusion criterion was children diagnosed with ASD and intellectual disabilities. An interdisciplinary team made the diagnoses using the criteria from the Diagnostic and Statistical Manual of Mental Disorders (DSM-5)^1^ and by performing the Brazilian version of the Autism Diagnostic Interview (ADI-R).^11^ All children have intellectual disabilities. A study conducted by the three university centers mentioned above details the full neuropsychological assessment.^12^ The Research Ethics Committee of UNIFESP approved this work.

The morphological physical examinations of the 62 patients, conducted by two clinical geneticists, were independently entered into a Microsoft Excel spreadsheet,Ó and each sign was classified as present or absent.

Miles’s^7^ proposed evaluation consists of 82 morphological characteristics that assess 12 body areas: stature; hair growth pattern; ear structure, size, and placement; nose size; face size and structure; philtrum; mouth and lips; teeth; hands; fingers and thumbs; nails; and feet. LDDB^8^ lists approximately two thousand characteristics categorized by codes. Clinical geneticists have been using this standardization of terminology for the past two decades, promoting a non-ambiguous list of definitions of these characteristics. All patients had their head circumference and stature measured, as well as the length of their ears, hands, and middle finger of the hands and feet. The point of the normal distribution of measurements was obtained with normal distribution curves by gender and age.^13^ Measurements two standard deviations below average were considered dysmorphic. The physical examination included audiovisual documentation.

We used Cohen’s Kappa coefficient to evaluate the agreement between the two observers.^14^ Agreement is a reliability measure, with values between 0.20 and 0.40 representing low agreement; between 0.4 and 0.6, moderate agreement; and greater than 0.6, high agreement.^14^

## RESULTS

A clinical geneticist, with more than five years of experience in care for patients with genetic syndromes, was the first to translate the 82 signs and a brief description of them;^7^ her native language is Portuguese, and she is fluent in English. Almost all signs were translated literally and accepted.

Strategies such as adding, omitting, and changing words and providing examples were used to explain the diverse educational and sociocultural realities of the Brazilian population, while ensuring that the words and expressions translated remained faithful to each specific situation measured by the original instrument (Table 1).^7^ For instance, the original English version exemplifies the description of the sign corresponding to the “dimpled or grooved chin” by comparing it to the chin of North American movie actor Kirk Douglas. This actor is not very well known in Brazil, and keeping the original description would make the interpretation of the sign difficult. For this reason, the expert committee agreed to describe the sign as “fovea mentalis (dimple) or a groove in the median region of the chin.”

**Table 1:** Description of morphological signs that underwent cultural adaptation in the translation from English into Portuguese.

Dysmorphic features	Brief description	Características dismórficas	Breve descrição
*Frontal upsweep*/*cowlick*	*Literally as if a cow had licked the frontal hair and it stayed in an upward sweep from the forehead*	Topete frontal	Cabelo levantado na parte superior da fronte
*Low-set ears*	*The topmost insertion of the ear is below a line drawn horizontally between the canthi of the eyes. Must assess with head upright*	Orelhas de baixa implantação	A inserção superior da orelha está abaixo de uma linha traçada horizontalmente entre o canto interno do olho e o ponto superior de inserção da orelha. Deve ser avaliada com a cabeça em posição vertical (paciente em vista frontal). Considerar também uma linha horizontal traçada entre o canto externo do olho e o ramo da hélice (paciente posicionado em vista lateral).
*Posteriorly rotated ears*	*Measure with head upright, ears rotated posteriorly >30°*	Orelhas pouco rodadas	Medida com a cabeça vertical, orelhas estão rodadas posteriormente ângulo >30° (paciente em vista lateral)
*Small ears/microtia*	*Small normal looking ears, or small dysplastic ears*	Orelhas pequenas/microtia	Orelhas pequenas de aspecto normal (microtia grau 1), ou orelhas pequenas e malformadas (microtia grau 2 ou 3)
*Crumpled ear helix*	*As if someone had crunched it between their thumb and fingers and the folds had stayed in place*	Hélice da orelha pregueada	Dobradura da hélice. Uma dobradura que compromete a hélix, antélice e tubérculo da orelha (como se essas estruturas fossem pinçadas entre o polegar e o indicador)
*Flat face*	*Subjective and overlaps with midface hypoplasia and flat malar region*	Face plana	Impressão subjetiva de face achatada podendo significar também hipoplasia do terço médio da face e hipoplasia da região zigomática
*Dimpled or grooved chin*	*A mid-line groove as in Kirk Douglas*	Mento com fosseta ou sulco	Uma fosseta (covinha) ou um sulco na região mediana do mento
*Large hands*	*Large, hypertrophied hands*	Mãos alongadas/alargadas	Mãos alongadas comprimento total da mão >2SD ou >percentil 97 ou alargadas (subjetiva)
*Small hands*	*Tiny, delicate, or thin hands*	Mãos pequenas	Minúsculas, delicadas ou mãos finas. Comprimento total da mão <2SD ou <percentil 3

In some items of the signs that describe ear structure, size, and placement, we were also careful to include the patient’s position in relation to the observer in the brief description of the morphological sign to describe it better. This example can be better understood in the item “low-set ears,” which the literal translation described as “The upper attachment of the ear is below a line drawn horizontally between the inner canthus of the eye and the upper point of attachment of the ear. The ear must be evaluated with the head in the upright position,” but the expert committee decided that, for a better understanding, the sign should be defined as follows: “The upper attachment of the ear is below a line drawn horizontally between the inner canthus of the eye and the upper point of attachment of the ear. The ear must be evaluated with the head in the upright position (patient in the frontal plane). A horizontal line between the outer canthus of the eye and the crus of the helix should also be considered (patient in the sagittal plane).”

Since the minor signs are subjective, the expert committee determined that the measurable signs that have specific tables^11^ according to age and gender should be used for better clinical interpretation, as in the description of hands and feet. “Large hands” would be literally described as is, but subjectively as “large/hypertrophied hands;” as this description is broad, we chose to define it as “elongated hands, total length of the hand >2 standard deviations or >97^th^ percentile or enlarged (subjective).” The same situation occurred in the item “small hands”, originally described as “tiny, delicate, or thin hands,” which, in addition to the original description, included the criteria established on the tables “tiny, delicate, or thin hands with total length of <2 standard deviations or <3^rd^ percentile.”

The expert committee standardized the 12 large body areas according to the *Nomina Anatomica*.^10^ As the use of anatomical terms is standardized, this measure should allow non-geneticist physicians from different Brazilian regions to understand the description of dysmorphic signs.

A Brazilian clinical geneticist who lives in the United States, has Portuguese as her native language, and is fluent in English performed the back translation. Naturally, the back translation was different in these cases, since the signs had already been culturally adapted and their descriptions changed for better understanding by non-specialist physicians.

The reference equivalence assessment was based on the comparison of terms and expressions of the original and back-translated versions in each of the questions. Each item was classified as similar, when the two versions were the same; close, when words or expressions were distinct, but without modifying the context; and different, when the literal meaning changed between the back-translated version and the original. Out of the 82 signs analyzed, 80 were considered similar (97.5%); 2, close (2.5%); and none was different. Based on this classification, we kept the changes made in the original text.

The general equivalence assessment took into account the result of the test. The items were reviewed, and the expert committee believes that part of the high error rate in some of these items was due to the low quality of the pictures used during the test or photos that were confusing as to the sign assessed. Their descriptions were reviewed, a new consensus was reached, and the final version was drawn up.

After the conclusion of the final version, two clinical geneticists performed the morphological physical examination in 62 patients with ASD on two separate occasions to verify the interobserver agreement for each item. Both examiners took all anthropometric measurements. We used the Kappa index to evaluate the agreement between the examiners. Among the 86 items assessed, 83 (96%) presented a Kappa index above 0.4 (most of them above 0.6), which represents a moderate to high interobserver agreement. Measurable items showed Kappa index=1. The observers disagreed in only one item (enamel abnormality, Kappa=-0.022), a fact that can be attributed to the difficulty of examining the teeth of children with ASD, who have an aversion to contact. The item “dysmorphic” and the 12 body areas presented high agreement between the observers. Table 2 shows the results of the Kappa index.

**Table 2:** Morphological examination indicated for children diagnosed with autism spectrum disorder. Agreement between two observers.

		Kappa index	p-value
1.	Short stature	1.0	<0.001
2.	Hair growth pattern	0.58	<0.001
3.	Ear structure, size, and placement	0.93	<0.001
4.	Nose size	0.93	<0.001
5.	Face size and structure	0.87	<0.001
6.	Philtrum	0.93	<0.001
7.	Mouth and lips	0.90	<0.001
8.	Teeth	0.62	<0.001
9.	Hands	1.0	<0.001
10.	Fingers and thumbs	0.36	<0,002
11.	Nails	0.81	<0,001
12.	Feet	0.88	<0.001
	Dysmorphic	0.78	<0.001

## DISCUSSION

The Brazilian literature has no studies to guide the investigation of morphological characteristics in individuals with autism or that have translated and culturally adapted published methods. In this regard, the present work is unique. It complements the availability of validated instruments since several studies have translated and culturally adapted psychometric scales.^8,15,16^

Translation and cultural adaptation^7^ utilize standardized methods and sensible criteria to produce adequate versions of foreign instruments for use in countries with different languages.^17^ This study followed the stages recommended by international guidelines,^9,11^ resulting in the development of the translated version of the instrument “Measure of dysmorphology: useful for autism subgroup classification,” which incorporates the Brazilian reality and culture. We expect that this work can help non-specialist physicians, in different regions of the Brazilian territory, to recognize minor signs and discrete phenotypic abnormalities, which in most cases go unnoticed and are present in many chromosomal microdeletion and microduplication syndromes. The lack of recognition of these signs by non-specialist physicians prevents the referral to a specialist, which can compromise the etiologic diagnosis and genetic counseling.

Flor et al.^18^ evaluated children born in North America and questioned whether there are differences among individuals classified as having essential autism and complex autism based on phenotypic assessments (cognitive functioning, adaptive behavior, the severity of autism, quality of life, and behavioral problems) and medical comorbidities. The study revealed that complex autism and essential autism can have distinct development profiles according to cognitive functioning, adaptive behavior, the severity of autism, quality of life, behavioral problems, and medical comorbidities. Thus, we emphasize the importance of searching for microcephaly and dysmorphic signs when evaluating children with ASD.

We concluded that the translated instrument meets all the international criteria, and minor anomalies and their clinical descriptions are standardized and recognizable. Such standardization will certainly help non-geneticist physicians to perform the physical examination in autistic children and determine whether or not they are dysmorphic. This outcome has direct implications in the causal investigation of this disorder.

Given the large extension of the country and the cultural and linguistic differences in each Brazilian region, a limitation of the present study is the fact that the expert committee consisted only of physicians who lived in the city of São Paulo. Some terms for describing dysmorphisms might not be the same in all Brazilian regions.

Our group is in the process of validating the proposed instrument^7^ to classify patients as dysmorphic and non-dysmorphic. In addition to various determinations, such as ADM sensitivity and specificity, we have obtained the frequency of morphological signs in children with typical development.^19^
